# Early adopters of paramedic prescribing: a qualitative study

**DOI:** 10.29045/14784726.2019.12.4.3.57

**Published:** 2019-12-01

**Authors:** Karen Stenner, Suzanne van Even, Andy Collen

**Affiliations:** University of Surrey; University of Surrey; College of Paramedics

## Abstract

**Aims:**

To explore the experience of paramedics who are early adopters of independent prescribing in a range of healthcare settings in the United Kingdom.

**Methods:**

Following a public consultation by NHS England in 2015, the decision was made in March 2018 to amend legislation to enable advanced paramedics to independently prescribe medicine in UK settings. Capturing the experience of these ‘early adopters’ will help to identify where paramedic prescribing can produce optimum benefits in healthcare systems, as well as enabling early scoping out of challenges to implementation and strategies for resolving challenges. This exploratory qualitative study involved interviews with 17 paramedics who have undertaken the independent prescribing programme in the United Kingdom. Participants were recruited via social media and regional paramedic networks between May and July 2019. Interviews were conducted by telephone or video call and explored use/anticipated use of prescribing, benefits and challenges to prescribing and support for the prescribing role. Thematic analysis was conducted to identify key themes.

**Results:**

Of the 17 participants, six were currently prescribing and the remainder were awaiting annotation. Participants worked in a range of settings, including: primary care, emergency departments, urgent care, walk in centres and rapid response services. Key benefits to prescribing were similar to those reported by other non-doctor prescribers and included: streamlining care for patients, improving safety, improving efficiency and facilitating new advanced clinical practice roles. Key challenges included: administrative IT issues, lack of ability to prescribe controlled drugs and managing patient/colleague expectations around paramedic prescribing. In general, participants felt supported in their prescribing role, both by doctors and other non-doctor prescribers, and felt confident to prescribe following the prescribing course. Concerns were raised about potential isolation in some settings, lack of parity in prescribing legislation across different professions and the way this is taught in prescribing programmes.

**Conclusion:**

Indications are that paramedic prescribing is rolling out successfully in line with expectations. Barriers and facilitators are similar to those reported by other non-doctor prescribers and independent prescribing is already an essential component to advanced practitioner roles in settings such as primary care. Findings highlight a need for greater alignment of prescribing legislation across non-doctor prescribers from different professions undertaking advanced roles.

